# *SUSD2* expression correlates with decreased metastasis and increased survival in a high-grade serous ovarian cancer xenograft murine model

**DOI:** 10.18632/oncotarget.27626

**Published:** 2020-06-16

**Authors:** Jordan N. Sheets, Mitch E. Patrick, Kristi A. Egland

**Affiliations:** ^1^Cancer Biology & Immunotherapies Group, Sanford Research, Sanford School of Medicine of The University of South Dakota, Sioux Falls, SD, USA; ^2^SAb Biotherapeutics, Sioux Falls, SD, USA

**Keywords:** high-grade serous ovarian cancer, SUSD2, xenograft, metastasis

## Abstract

The cause of death among high-grade serous ovarian cancer (HGSOC) patients involves passive dissemination of cancer cells within the peritoneal cavity and subsequent implantation of cancer spheroids into adjacent organs. *Sushi Domain* *Containing 2* (*SUSD2*) encodes a type I transmembrane protein containing several functional domains inherent to adhesion molecules. Previous studies using *in vitro* methods have indicated that *SUSD2* functions as a tumor suppressor in several cancers, including HGSOC. In this study, we generated a HGSOC xenograft mouse model to investigate *SUSD2* expression in the context of HGSOC late-stage metastasis and overall survival. OVCAR3 cells with knock-down expression of *SUSD2* (OVCAR3 SUSD2-KD) or endogenous expression of *SUSD2* (OVCAR3-Non-Targeting (NT)) were injected into the peritoneal cavity of athymic nude mice. Immunohistochemistry analysis was utilized to identify infiltrating cancer cells and metastatic tumors in mouse ovaries, pancreas, spleen, omentum and liver. OVCAR3-NT mice developed significantly less cancer cell infiltrate and tumors in their pancreas and omentum compared to OVCAR3 SUSD2-KD mice. Furthermore, OVCAR3-NT mice displayed a longer median survival when compared to OVCAR3 SUSD2-KD mice (175 days and 185.5 days, respectively; *p*-value 0.0159). Altogether, the findings generated through the preclinical mouse model suggest that increased *SUSD2* expression in HGSOC impedes *in vivo* metastasis to pancreas and omentum. These results correlate to longer median survival and prove to be consistent with previous findings showing prolonged survival of HGSOC patients with high *SUSD2*-expressing primary tumors.

## INTRODUCTION

Epithelial ovarian cancer (EOC) is the leading cause of death in gynecological malignancy and ranks 5th in mortality rates among all cancers in the United States [1]. EOC is accompanied by vague and minor symptoms during onset and initial stages of the disease progression (stage I-II), which impedes patient diagnosis at an early stage [2, 3]. Unfortunately, approximately 75% of EOC patients are diagnosed when the cancer has become widely disseminated in the peritoneal cavity (stage III-IV) [2, 4]. The American Cancer Society estimates that 13,980 women will die from EOC in 2019 (representing ~62% of women initially diagnosed with ovarian cancer) [1], showing the need for early detection tools and novel approaches to treatment. The majority of EOC cases are diagnosed as high-grade serous ovarian carcinomas (HGSOC), representing the most deadly of all EOC subtypes [5].

SUSD2 is a type I transmembrane protein that contains a somatomedin B, adhesion-associated domain present in MUC4 and other proteins (AMOP), von Willebrand factor type D and Sushi domains, which frequently are found in molecules associated with cell-cell and cell-matrix adhesion. SUSD2 has a predicted molecular weight of 90.4 kDa (Supplementary Figure 1). However, N-linked glycosylation can occur at nine potential asparagine residues predicting a mature protein of 100–110 kDa [6]. Previous western immunoblot analysis utilizing antibodies targeting epitopes in the C-terminus of SUSD2 or its mouse homologue mSVS-1, showed 2 predominant bands of approximately 110 kDa and 60 kDa in size [6, 7]. This shorter polypeptide suggested that SUSD2 is post-translationally cleaved near the middle of the protein sequence. Fluorescent western immunoblot using antibodies directed against the N-terminal and C-terminal fragments of SUSD2 revealed a 50 kDa band alongside the previously observed 60 kDa C-terminal SUSD2 fragment and 110 kDa full length polypeptide [8]. Consistent with the size of SUSD2 bands observed by western immunoblot analysis, Edman degradation and mutagenesis studies of SUSD2 identified the cleavage site between the aspartic acid (452) and proline (453) residues of SUSD2’s GDPH sequence in the von Willebrand factor domain [8].

The mouse homolog of SUSD2 (susd2/mSVS-1) was first studied in 2007 by Sugahara and colleagues, demonstrating that overexpression of *susd2* in HT1080 fibrosarcoma cells and HeLa cervical carcinoma cells correlated with decreased clonogenicity, anchorage-independent growth, migration, and invasion through matrigel, implicating susd2 as a potential tumor suppressor [9]. Since then, several publications have begun to explore *SUSD2* expression in the context of multiple cancers including ovarian [7], breast [6, 10], lung [11–13], renal [11, 12], gastric [14], liver [15], and colon [16].

*In vitro* assays and clinically annotated tissue microarrays (TMAs) collectively support the notion that SUSD2 may function as a tumor suppressor in ovarian, renal, lung, liver, and colon cancer, although the exact mechanism of tumor suppression remains to be elucidated [7, 11–13, 15, 16]. Additionally, data archived in The Cancer Genome Atlas (TCGA) suggested that HGSOC patients with amplified copy numbers of SUSD2 survive longer than HGSOC patients with unaltered copy numbers of SUSD2. However, it should be noted that reduced sampling size limited the statistical analysis of this correlation. In a recent study, our laboratory analyzed the function of SUSD2 in HGSOC by performing immunohistochemical (IHC) analysis of HGSOC tissue microarrays using an anti-SUSD2 antibody. The Egland Lab showed that higher levels of SUSD2 in primary tumors correlated with an increased median survival in HGSOC patients [7]. In addition, we generated *in vitro* models to study the functions of SUSD2 in HGSOC by using the OVCAR3, KURAMOCHI and OVSAHO cells lines. These cell lines were chosen because they are genotypically similar to HGSOC patient tumors [17]. Decreased *SUSD2* expression in OVCAR3, KURAMOCHI and OVSAHO cells increased cell migration and up-regulated many well characterized genes coding for Epithelial-Mesenchymal Transition (EMT) proteins [7]. *In vitro* mesothelial clearance assays demonstrated that decreased *SUSD2* expression in OVCAR3 and KURAMOCHI spheroids increased the efficiency of mesothelial clearance [7]. Conversely, the role of SUSD2 in breast cancer may be tumorigenic in nature; overexpression of *SUSD2* in MDA-MB-231 cells has been shown to increase invasion through matrigel and induce T cell apoptosis in co-culture experiments [6]. A recent study of SUSD2 in breast cancer patients has implicated SUSD2 in the recruitment of tumor associated macrophages by inducing increased cancer cell secretion of Monocyte Chemoattractant Protein-1 (MCP-1) [10]. Together, these findings suggest multiple functions of SUSD2 that may depend on the type of cancer and/or microenvironment.

In the present study, we generated an OVCAR3 athymic nude mouse model to investigate the role of SUSD2 in late-stage metastasis of HGSOC. Because previous *in vitro* analysis confirmed that SUSD2 inhibits mesothelial clearance in HGSOC cells, we hypothesized that decreased *SUSD2* expression in HGSOC cells increases the overall tumor burden and contributes to shorter survival.

## RESULTS

### Characterization of HGSOC SUSD2 knock-down and control cell lines

**Figure 1:**
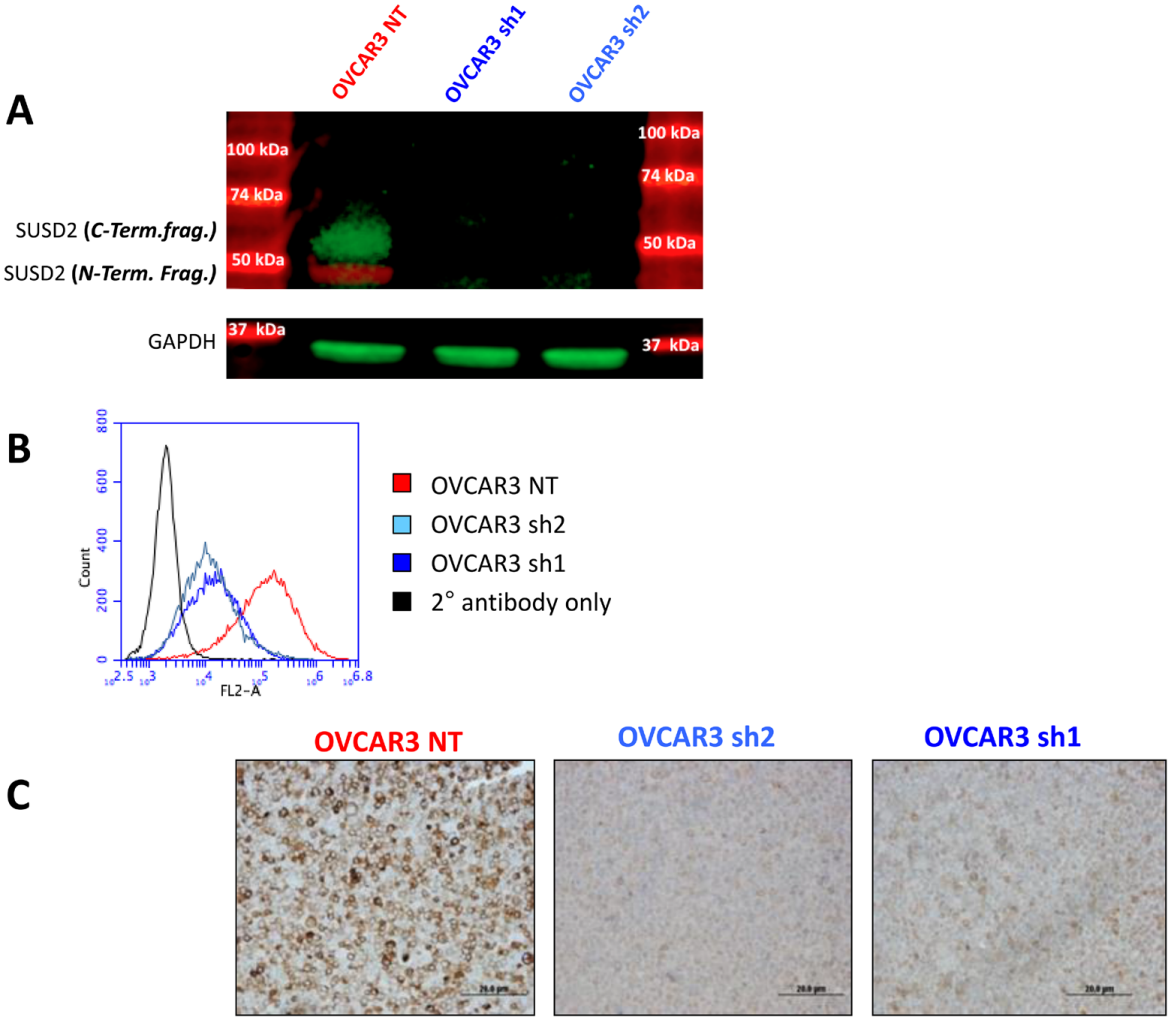
Characterization of non-targeting and SUSD2 knock-down sh1 and sh2 OVCAR3 cell lines. (**A**) Analysis of SUSD2 polypeptides in OVCAR3 whole cell lysates. OVCAR3 was transfected with either a non-targeting (NT) shRNA construct or *SUSD2*-targeting shRNA constructs (sh1, sh2). The image shows fluorescent protein bands (green and red) from western immunoblot analysis. Antibodies were used to detect the N-terminal (red band, ~50 kDa) and C-terminal (green band, ~65 kDa) fragments of SUSD2. The lower panel shows GAPDH bands from whole cell lysates used as a control. (**B**) Flow cytometry analysis was performed on OVCAR3 cells using an anti-SUSD2 antibody. OVCAR3 was transfected with either a non-targeting shRNA construct (NT; shown in red) or one of two *SUSD2*-targeting shRNA constructs (sh1 and sh2; shown in shades of blue). The black histograms in each graph depicts the secondary antibody alone. Fluorescence is shown in the x-axis and number of cells is shown in the y-axis. (**C**) IHC staining of SUSD2 in cell pellets from OVCAR3 NT and *SUSD2* knock-down sh1 and sh2 cell lines. Paraffin-embedded cell pellets were sectioned and stained using an anti-SUSD2 antibody. The brown color indicates positive SUSD2 staining.

To investigate the metastatic consequences of *SUSD2* expression in HGSOC, we utilized a previously characterized HGSOC cell line, OVCAR3. Because OVCAR3 cells endogenously express *SUSD2*, knock-down and control cell lines were generated. Short hairpin RNAs, either non-targeting (NT) or SUSD2-targeting, were transfected into the OVCAR3 parental cell line. After selection and characterization of clones, stable cell lines were established including OVCAR3-NT and stable OVCAR3-SUSD2-knock-down (KD) cell lines, OVCAR3 sh1 and OVCAR3 sh2. Western immunoblot analysis was conducted using two separate anti-SUSD2 antibodies; one antibody targeting the N-terminal fragment of SUSD2, and the other targeting the C-terminal fragment of SUSD2. Results showed ~60 kDa and ~50 kDa bands present only in the OVCAR3-NT whole cell lysates, verifying appropriate endogenous and SUSD2-KD expression in the generated HGSOC cell line (Figure 1A). Endogenous SUSD2 is localized on the cell surface. To confirm both localization and amount of SUSD2 at the cell membrane of OVCAR3 cells, flow cytometry analysis was conducted on non-permeabilized cells. OVCAR3-SUSD2-KD cell line had lower levels of SUSD2 compared to the NT control (Figure 1B). IHC analysis was utilized to confirm western immunoblot and flow cytometry analyses, demonstrating decreased positive staining for SUSD2 in cell pellets composed of OVCAR3 SUSD2-KD cells compared to the OVCAR3-NT control (Figure 1C).

### OVCAR3 SUSD2-KD mice correlated with increased tumor burden at initial time of sacrifice

**Figure 2:**
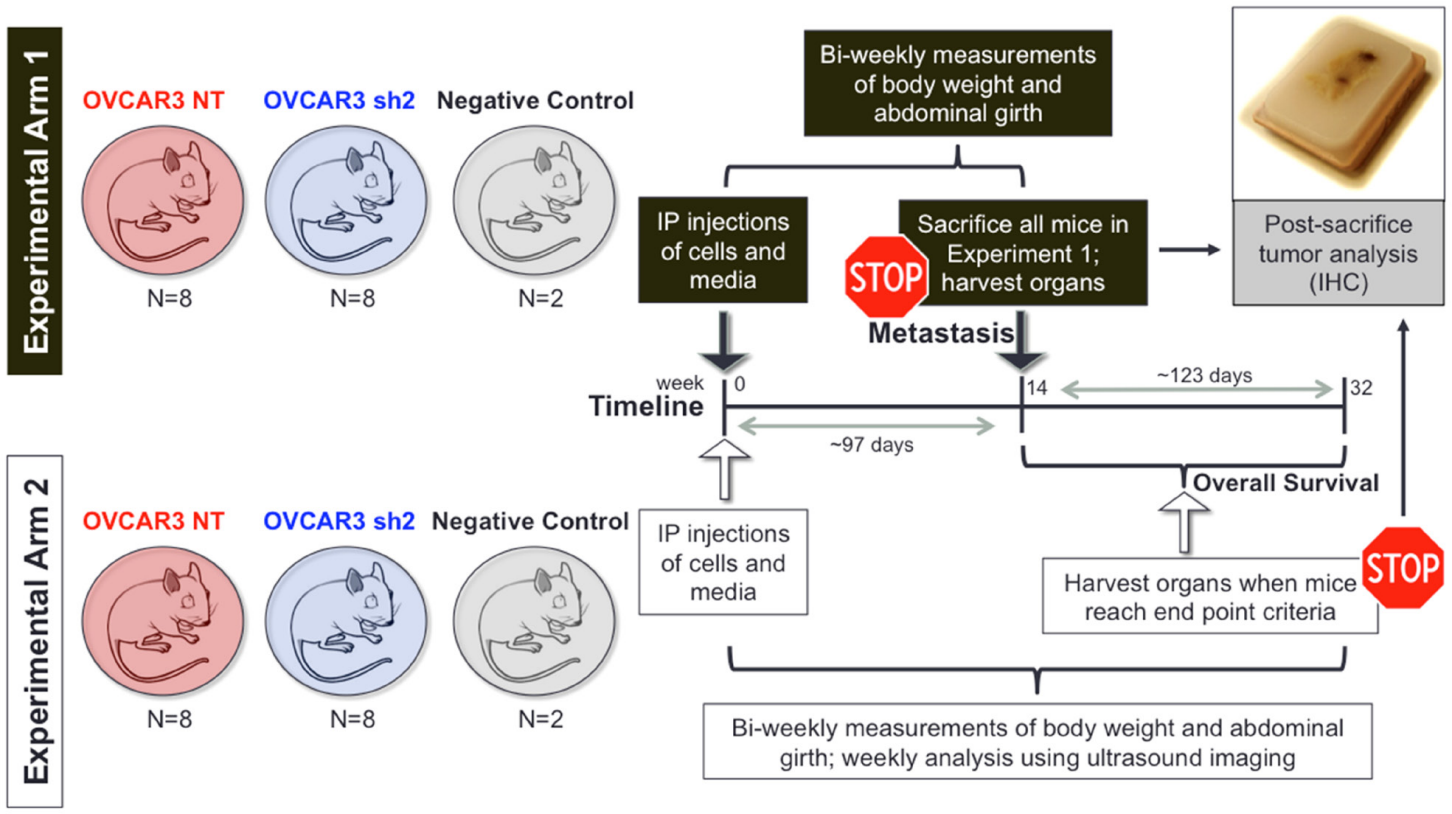
*In vivo* experimental design used to investigate the role of SUSD2 in HGSOC late-stage metastasis and overall survival. Work-flow for the athymic nude mouse model. Athymic nude mice (6-8 week-old females) received intraperitoneal (i.p.) injection of OVCAR3-NT cells (cells with endogenous expression of *SUSD2*), OVCAR3 sh2 cells (cells with knock-down expression of *SUSD2*), or serum free media (negative controls). *Experimental Arm 1* was designed to investigate the role of SUSD2 in early HGSOC metastasis (defined as 97 days or ~14 weeks post i.p. injection). *Experimental Arm 2* was designed to investigate the role of SUSD2 in overall survival related to HGSOC. Both experimental arms utilized ultrasound biomicroscopy (UBM) as a non-invasive method to visualize nodules in various organs within the peritoneal cavity during HGSOC progression. Murine pancreata, omentum, liver, ovaries, and spleen were harvested at 14 weeks post i.p. injection (*Exp. Arm 1*) or upon mice reaching preset end-point criteria (*Exp. Arm 2*).

To investigate the role of SUSD2 in HGSOC metastasis and overall survival *in vivo*, we generated a xenograft mouse model. The cell lines selected for use in this model were OVCAR3-NT and OVCAR3 sh2 of the SUSD2-KD cell lines (Figure 1). A total of 36 female athymic nude mice were injected via the intraperitoneal (i.p.) route with OVCAR3-NT cells (*n* = 16), OVCAR3 sh2 cells (*n* = 16), or serum free media control group (*n* = 4); time of OVCAR3 inoculation is illustrated “0 weeks” in Figure 2). Groups of mice (OVCAR3-NT mice, OVCAR3 sh2 mice, and control mice) were further divided into 2 experimental arms (*Experimental Arm 1* and *Experimental Arm 2*, Figure 2). Each experimental arm consisted of 1 group of OVCAR3-NT mice (*n* = 8), 1 group of OVCAR3 sh2 mice (*n* = 8), and 1 group of control mice (*n* = 2). All mice were assessed bi-weekly for HGSOC progression post-inoculation utilizing non-invasive measurements, such as body weight, abdominal girth, and ultrasound biomicroscopy (UBM).

**Figure 3:**
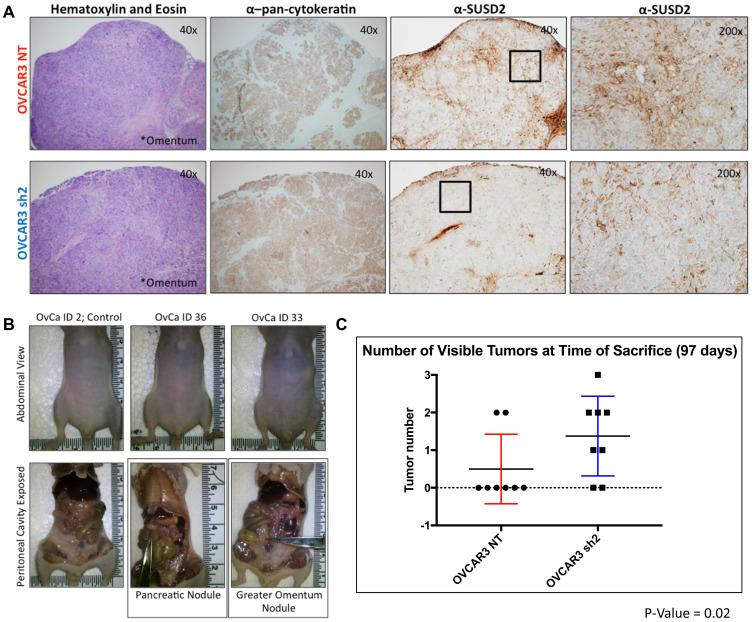
SUSD2-KD mice from *Experimental Arm 1* had increased tumor formation. (**A**) IHC analysis of gross tumors utilizing H&E, cytokeratin and SUSD2 staining at 40×. Images of SUSD2 staining at 100× are also shown (right column). Black box in 40× SUSD2 images indicate area used for 100× SUSD2 images. (**B**) Gross analysis of visible tumors. The scissors in the two images shown in the lower panel indicate pancreatic and omentum tumors. (**C**) Number of visible tumors at initial time of sacrifice. The graph depicts the total number of gross tumors for each mouse analyzed from each cohort (*n* = 8).

The goal of *Experimental Arm 1* was to assess the role of SUSD2 in HGSOC metastasis. Mice were sacrificed at 14 weeks post-inoculation for assessment of metastatic disease. A pilot experiment demonstrated that 14 weeks was a suitable time to assess early metastatic disease in our model (data not shown). At the time of sacrifice, no significant differences were observed in mouse weight, girth or ascites formation between OVCAR3-NT mice and OVCAR3 sh2 mice (Supplementary Figure 2A–2C). IHC analysis was performed using both anti-pan-cytokeratin and anti-SUSD2 antibodies to visualize tumor cells and contrast the level of SUSD2 in tumors from each cohort of mice (Figure 3A). Tumors were observed in the omentum and pancreas of both OVCAR3-NT and OVCAR3 sh2 at initial time of sacrifice (Figure 3B). However, OVCAR3-NT mice displayed an average of 0.5 tumor; whereas, OVCAR3 sh2 mice displayed an avg. of 1.38 tumors, *p*-value = 0.02 (Figure 3C).

**Figure 4:**
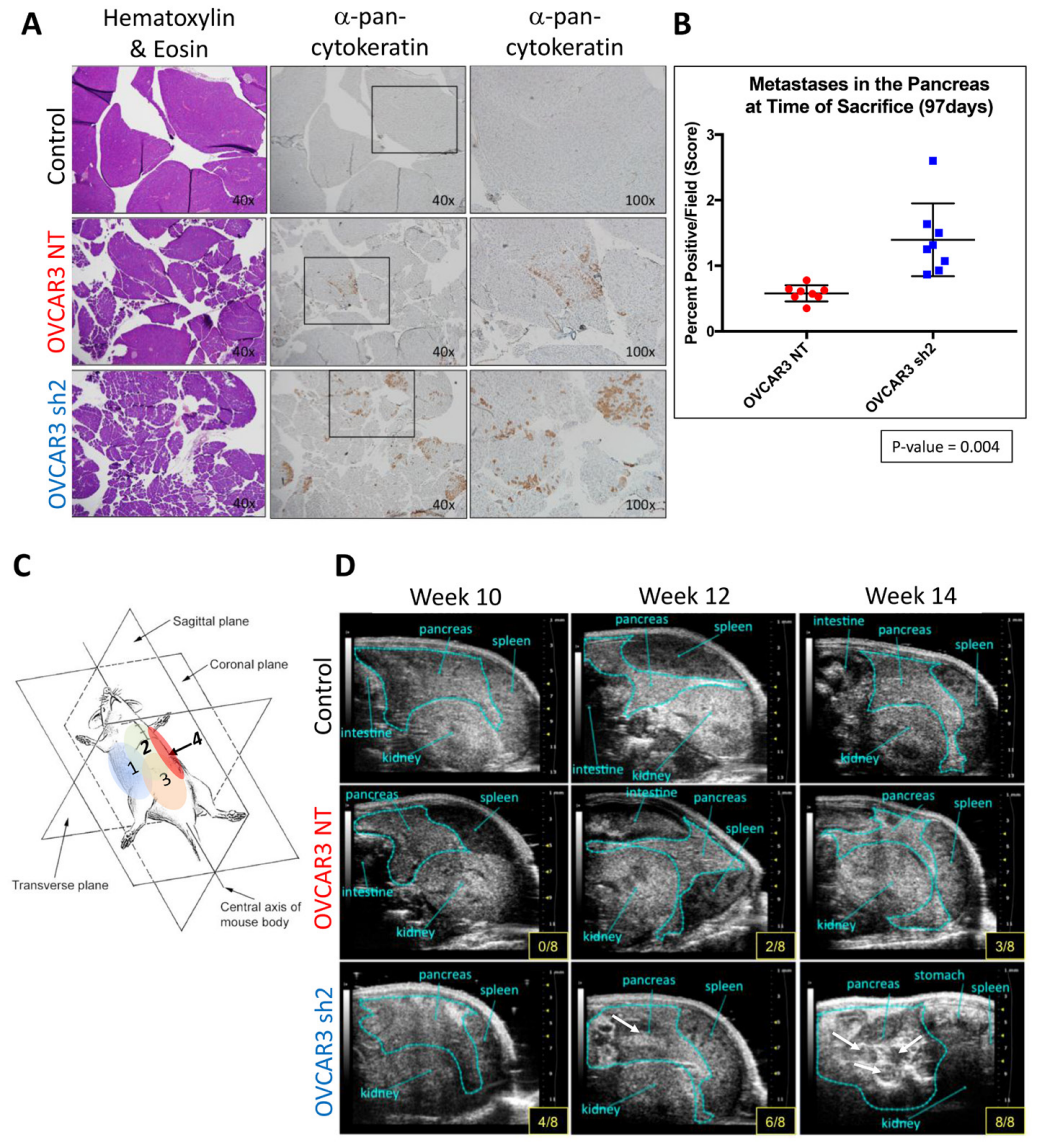
SUSD2-KD mice showed increased pancreatic tumor burden at initial time of sacrifice. (**A**) Representative images of H&E staining and pan-cytokeratin staining from each group of mice are shown at varying magnifications. Black boxes outline areas of tissue that were imaged under higher magnification (right column). (**B**) Quantification of pancreatic cancer cell infiltrate presented as percent of positive staining/field of view. (**C**) Illustration of the orientation of the mouse during UBM imaging. Each quadrant is labeled on the mouse’s abdomen, 1–4; ultrasound images shown in D were taken from quadrant 4, shown in red. (**D**) Ultrasound images depict the progression of tumor formation in the peritoneal cavity, from weeks 10–14 post i.p. injection of OVCAR3 cells. The light blue outline in each image highlights the 2-D area of the pancreas. Each panel shows the number of mice that have displayed visible cysts/tumors (white arrow) at that time point (bottom right of each panel).

To assess the microscopic metastases in mouse pancreata at initial time of sacrifice, entire tissue sections of pancreas harvested from each mouse in *Experimental Arm 1* were analyzed for cytokeratin staining via IHC (Figure 4A) Supplementary Figure 3 defines the percent positive per field score for the tissue sections, such that the extensiveness of stain is represented by 0 to 9. The area of field occupied by tissue was also accounted for with a scale from 0.25 to 1. The use of these scores to determine surface area occupied by tumor is detailed in the methods section. OVCAR3-NT mice displayed less visible tumors and showed less microscopic cancer cell infiltrate when compared with the OVCAR3 sh2 cohort; OVCAR3-NT mice had ~7.5% of pancreas surface area occupied by tumor cells compared to ~12.13% in OVCAR3 sh2 mice, *p*-value = 0.004 (Figure 4A and 4B). Furthermore, UBM analysis of mice revealed cyst/tumor formation occurring earlier in the OVCAR3 sh2 mice when compared to the OVCAR3-NT mice (Figure 4C and 4D). At week 10 post-injection, UBM analysis of quadrant 4 of the peritoneal cavity showed pancreata from the control group displaying the stereotypical and consistent patterning that is attributed to a healthy pancreas (Figure 4D). The pancreata of OVCAR3-NT mice were similar to control pancreata without visible cysts/tumors; whereas half of the OVCAR3 sh2 mice showed lensing or dark splotches in the pancreas, indicating cyst or tumor formation, respectively (Figure 4D). Upon time of sacrifice (week 14), UBM analysis indicated 1 or more tumors present in the pancreas of every OVCAR3 sh2 mouse; however, only 3 mice from the OVCAR3-NT group displayed tumor formation at 14 weeks (Figure 4D, yellow boxes in the lower right-hand corner of the images).

**Figure 5:**
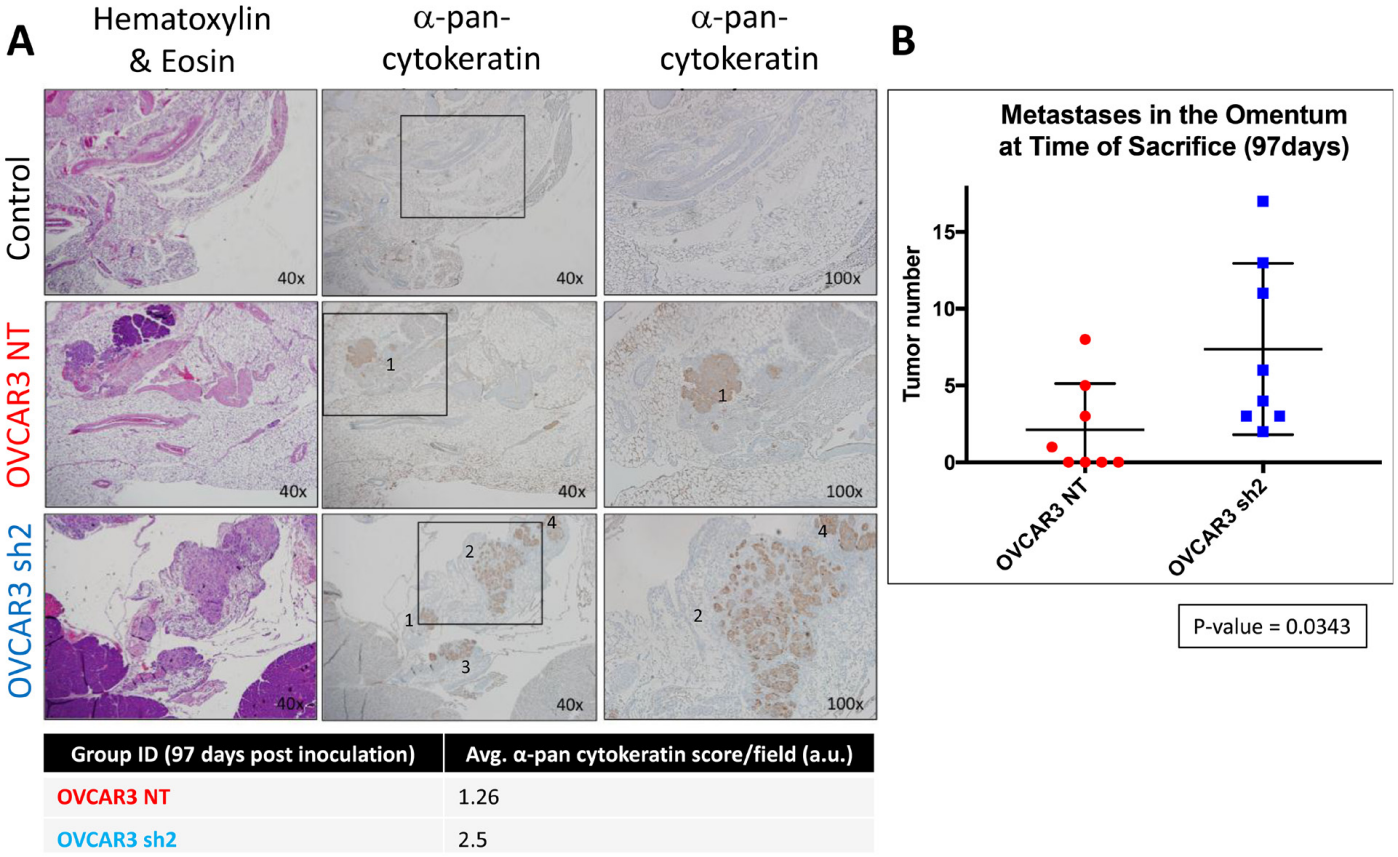
SUSD2-KD mice showed increased tumor burden in the omentum at initial time of sacrifice. (**A**) Representative images of H&E and pan-cytokeratin staining from each group of mice are shown. Black boxes outline areas of an image that were viewed under higher magnification and reimaged (right column). Black numbers displayed over positive pan-cytokeratin staining indicate a nodule within the omentum. The table below the images lists the avg. score/field of positive immunostaining for pan-cytokeratin in the omentum of mice 97 days post-inoculation. (**B**) Quantification of nodules in the omentum. The total number of nodules from each mouse (*n* = 8) is depicted in the graph.

At initial time of sacrifice, tissue sections of omentum were harvested from each mouse in *Experimental Arm 1* and stained via IHC for cytokeratin using an anti-cytokeratin antibody to assess the microscopic metastases in mouse omentum (Figure 5A). OVCAR3-NT mice displayed less visible tumors and nodules (microscopic aggregates of OVCAR3 cells that stained positive for cytokeratins) when compared with the OVCAR3 sh2 cohort. An average of 2.34 nodules was observed in OVCAR3-NT mice, and an average of 7.12 nodules was observed in OVCAR3 sh2 mice, *p*-value = 0.03 (Figure 5B). Altogether, our findings gathered from *Experimental Arm 1* showed that knock-down of SUSD2 in OVCAR3 cells contributes to higher overall tumor burden in a HGSOC mouse model.

### OVCAR3-NT mice lived significantly longer than OVCAR3 knock-down mice

**Figure 6:**
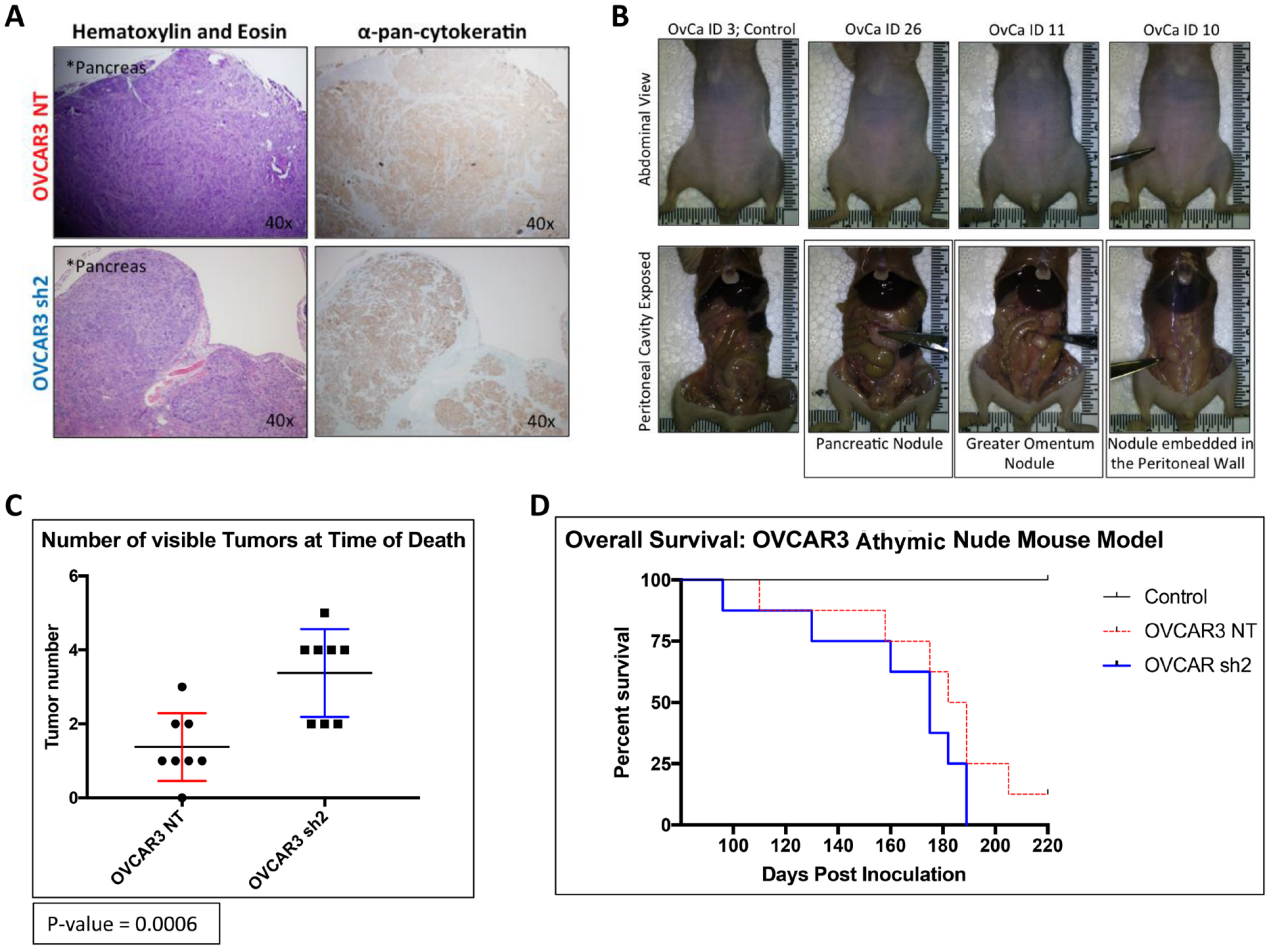
OVCAR3-NT mice lived significantly longer than OVCAR3 SUSD2-KD mice. (**A**) IHC analysis of gross tumors at time of death using H&E and pan-cytokeratin staining. Magnification of images is indicated in the top right-hand corner of each image. (**B**) Images of murine peritoneal cavity before harvesting tumors. Images are displayed as a paired set; each picture in the top panel correlates with the picture directly below it, displaying the mouse’s abdomen before dissection (top) and the peritoneal cavity after dissection (bottom). Scissors point to gross tumors visible before and after dissection of the peritoneal cavity. (**C**) Quantification of gross tumors at time of death. The total number of tumors from each mouse (*n* = 8) is depicted in the graph. (**D**) Overall Survival of mice. The Kaplan–Meier curve shows the number of days that the mice survived post i.p. injection of OVCAR3 cells (*n* = 8 for both OVCAR3 cohorts, *n* = 2 for the control group).

The goal of *Experimental Arm 2* was to assess the role of SUSD2 in overall survival associated with HGSOC progression. The parameters of *Experimental Arm 2* were virtually the same as *Experimental Arm 1* with the exception of the time of sacrifice (Figure 2). Mice from *Experimental Arm 2* were not sacrificed simultaneously as mice from *Experimental Arm 1* but were sacrificed upon each individual mouse reaching pre-designated end point criteria (see Materials and Methods). Mice were monitored for HGSOC progression bi-weekly using non-invasive measurements (body weight, girth and UBM), and upon sacrifice, mouse tissues were harvested and analyzed as previously described for mice in *Experimental Arm 1*. Pancreata from both OVCAR3-NT and OVCAR3 sh2 mice showed tumor formation with positive pan-cytokeratin staining (Figure 6A). Figure 6B shows identification of visible tumors at the time of sacrifice. The OVCAR3 sh2 cohort displayed significantly more tumors than OVCAR3-NT mice. OVCAR3-NT mice exhibited an average of 1.375 tumors compared to an average of 3.375 tumors in OVCAR3 sh2 mice, *p*-value = 0.0006 (Figure 6C). OVCAR3-NT mice survived for an average of 10.5 days longer than OVCAR3 sh2 mice with a median survival of 185 days for OVCAR3-NT mice compared to 175 days for OVCAR3 sh2 mice, *p*-value 0.0159 [Gehan-Breslow-Wilcox test]) (Figure 6D).

Once defined endpoints for *Experimental Arm 2* were reached, the pancreas was harvested from each mouse. To assess the microscopic metastases, entire tissue sections of pancreas were analyzed for cytokeratin staining via IHC (Supplementary Figure 4A). OVCAR3-NT mice displayed fewer visible tumors in the pancreas than OVCAR3 sh2 mice. The pancreas of OVCAR3-NT mice displayed a total of 10 tumors; whereas, OVCAR3 sh2 mice displayed a total of 19 tumors (Supplementary Figure 4C). Unexpectedly, when the entire pancreata was surveyed microscopically, the total cancer cell infiltrate/pancreas was observed to be the same between OVCAR3-NT mice and OVCAR3 sh2 mice. Approximately 28.45% of the OVCAR3-NT pancreas surface area was occupied by cancer cells, and ~30.94% of OVCAR3 sh2 pancreas surface area was occupied by cancer cells, *p*-value = 0.41). (Supplementary Figure 4B). OVCAR3-NT mice had significantly less visible tumors in the pancreas compared to OVCAR3 sh2 mice, yet both groups showed a similar percentage of pancreas surface area occupied by cancer cells. Therefore, we investigated the relative size of tumors in each cohort of mice at time of death. ImageJ software was utilized to quantify the 2-D surface area of all pancreatic tumors previously imaged from IHC analysis, revealing that OVCAR3-NT mice contained significantly larger tumors than OVCAR3 sh2 mice. The average area of OVCAR3-NT tumor was 21.8 × 10^4^ a. u.^2^ compared to the average area of OVCAR3 sh2 tumor of 12.81 × 10^4^ a. u.^2^, *p*-value = 0.002) (Supplementary Figure 4D).

To assess the microscopic metastases in the omentum at the time of death from Experimental Arm 2 mice, entire tissue sections of omentum were harvested from each mouse and analyzed by cytokeratin staining via IHC (Supplementary Figure 5). Only 1 visible tumor was observed in the omentum of an OVCAR3 sh2 mouse, and the microscopic analysis of tumor infiltrate showed no significant differences between OVCAR3-NT or OVCAR3 sh2 mice. OVCAR3-NT mice had an average of ~10.05% of omentum occupied by tumor cells, and OVCAR3 sh2 mice had an average of ~14.15% of omentum occupied by tumor cells, *p*-value = 0.41. Consistent with *Experimental Arm 1*, *Experimental Arm 2* showed that knock-down expression of SUSD2 in a HGSOC OVCAR3 mouse model contributes to increased tumor formation. *Experimental Arm 2* allowed for analysis of overall survival and showed that knock-down of SUSD2 decreased overall survival.

## DISCUSSION

Almost 80% of HGSOC patients are diagnosed with distant metastatic disease (FIGO III-A, III-B, III-C, III-NOS, IV) at presentation, and the current 5-year survival rate of patients with metastatic HGSOC in the United States is about 32% [18]. Findings from our previous study demonstrated that high levels of SUSD2 in the primary tumor of HGSOC patients predicts increased patient survival (patients with high levels of SUSD2 in their primary tumor lived an average of 18 months longer than patients with low levels of SUSD2 in their primary tumor; Wilcoxon’s transformed *P*-value = 0.032). This suggested that SUSD2 may function as a tumor suppressor and prognostic marker in HGSOC patients with late-stage diagnosis [7]. Moreover, RT-qPCR analysis and *in vitro* mesothelial clearance assays demonstrated that decreased *SUSD2* expression in OVCAR3 and KURAMOCHI cell lines correlated with upregulation of genes coding for well-characterized EMT proteins and promotion of mesothelial clearance [7]. EMT is implicated in early-stage HGSOC metastasis when transformed cells begin to lose their cell-cell adherence and become more motile, contributing to passive exfoliation [19, 20]; whereas, mesothelial clearance is implicated in late-stage HGSOC metastasis, regulating the efficiency of spheroid implantation into the peritoneal wall [21, 22]. Altogether, these findings suggest that SUSD2 may play an important role in regulating early and late-stage HGSOC metastasis. In this study, we expand upon our previous work by testing the hypothesis that increased *SUSD2* expression inhibits HGSOC metastasis.

A recent study by Xu *et al*. suggested that SUSD2 promotes cancer metastasis in HGSOC [23]. However, the cell lines used for this study have been shown to poorly represent HGSOC. SKOV3 cells have been found to be highly unlikely to be HGSOC [17], and HO8910 cells have been shown to actually be a derivative of or contaminated with HeLa cells, a cervical cancer line [24]. To investigate the role of SUSD2 in HGSOC metastasis *in vivo*, we generated a xenograft mouse model by injecting human OVCAR3 cells as single-cell suspension directly into the peritoneal cavity of athymic nude mice (Figure 2). We chose to inoculate our xenograft mouse model with OVCAR3 cells (OVCAR3-NT and OVCAR3 sh2 cells) for multiple reasons (Figure 2). First, OVCAR3 xenograft mouse models have been shown to recapitulate tumor heterogeneity via recruitment of host stromal cells and maintenance of both tumor stroma and tumor/non-tumor vasculature interactions [25]. Furthermore, among the various HGSOC cell lines used in the literature today, the OVCAR3 cell line remains the most widely characterized, proven to accurately represent HGSOC both genetically and histologically [17, 26]. Moreover, the OVCAR3 cell line was derived from patient ascites [27, 28], representing an accurate phenotype of HGSOC cells immediately after early-stage, passive exfoliation, while still allowing for recapitulation of HGSOC late-stage metastasis in an i.p. xenograft mouse model. And lastly, OVCAR3 cells have been successfully passaged in mice and deemed suitable for the development of an i.p. xenograft mouse model, demonstrating wide-spread dissemination across multiple organs residing in the peritoneal cavity [25].

Upon initial time of sacrifice (14 weeks post inoculation of OVCAR3 cells), liver, pancreas, ovaries, omentum, and spleen were harvested from all mice from *Experimental Arm 1* (Figure 2). IHC analysis of tissues from each organ revealed that HGSOC metastasis was limited to the omentum and the pancreas. OVCAR3-NT mice had significantly less overall tumor burden (visible tumors and cancer cell infiltrate) in both the omentum and the pancreas when compared to the OVCAR3 sh2 mice (Figures 3–5). As a non-invasive method to track the progression of tumors in the peritoneal cavity of mice, we utilized UBM. Consistent with the IHC analysis of mouse pancreas, UBM demonstrated earlier cyst and/or tumor formation in the pancreata of OVCAR3 sh2 mice when compared with the OVCAR3-NT mice (Figure 4D). These results suggested that decreased *SUSD2* expression in HGSOC may increase metastasis to omentum and pancreas, which is consistent with our previous findings showing that decreased *SUSD2* expression inhibits mesothelial clearance [7].

Mice from *Experimental Arm 2* were sacrificed upon end point criteria to investigate if SUSD2 affected overall survival (Figure 2). OVCAR3-NT mice displayed a modest, yet significant increase in median survival when compared to the OVCAR3 sh2 mice (175 days vs. 185.5 days, respectively; *p*-value = 0.005 [Matnel-Cox]; Figure 6D), suggesting that decreased *SUSD2* expression in HGSOC predicts decreased overall survival, consistent with our metastatic analysis (IHC and UBM analyses) performed on mice from *Experimental Arm 1.* IHC analysis was also performed on mouse organs harvested from mice in *Experimental Arm 2*, demonstrating an increase in overall tumor burden when compared to that observed in mice from *Experimental Arm 1*. *Experimental Arm 2* showed an additional site of metastasis, the peritoneal wall (Figure 6B). The additional tumor burden and metastatic site is likely explained by the increased HGSOC progression associated with longer incubation before sacrifice. In *Experimental Arm 2,* OVCAR3 sh2 mice were shown to harbor almost twice as many pancreatic tumors as OVCAR3-NT mice; however, cytokeratin staining of entire pancreata showed no difference in total tumor surface area per pancreas between OVCAR3-NT mice and OVCAR3 sh2 mice (Supplementary Figure 4C). Utilizing Image J software, close examination of IHC slides of pancreatic tumors was performed, showing that the average size of tumor residing in the pancreas of OVCAR3-NT mice was significantly greater when compared to pancreatic tumor in OVCAR3 sh2 mice (Supplementary Figure 4C). The larger pancreatic tumor size of OVCAR3-NT mice explains why there was no difference in total tumor surface area despite OVCAR3 sh2 mice having more pancreatic tumors. This discrepancy in tumor size could be explained by the longer overall survival of OVCAR3-NT mice, thus allowing tumors to grow for a longer time period than those of OVCAR3 sh2 mice. Taken together, these findings indicate that SUSD2 is associated with decreased numbers of pancreatic metastases.

The contrasting phenotypes of SUSD2 in breast and other epithelial cancers may be explained by differences in metastasis mechanisms [29, 30], microenvironments, subcellular localizations of SUSD2, or splice-form variants of *SUSD2*. However, the results from this study suggest that SUSD2 acts as a tumor suppressor through the inhibition of late-stage metastasis in HGSOC. Although the exact mechanism of action remains unknown, results from our animal model suggested that SUSD2 inhibits metastatic spread of ovarian cancer in the abdominal cavity, consistent with findings from our previous study showing that SUSD2 may decrease mesothelial clearance. Future studies investigating how *SUSD2* expression in HGSOC may impact other factors that affect patient outcome, such as chemotherapy resistance of spheroids or the ability of the cancer cells to evade the immune system, will increase our understanding of the dynamic phenotypes of SUSD2 in cancer. In addition, determining the protective function of SUSD2 in HGSOC tumors may uncover useful knowledge in the development of novel therapies to improve survival of HGSOC patients.

## MATERIALS AND METHODS

### Cell culture

OVCAR3 cell lines were purchased from ATCC (Manassas, VA, USA) and maintained in DMEM with 10% fetal bovine serum (Atlanta Biologicals, Flowery Branch, GA, USA), 1 μg/ml puromycin and grown at 37°C in humidified 5% CO_2_. KURAMOCHI cell lines were a generous gift from the Drapkin Laboratory. KURAMOCHI cell lines were maintained in RPMI with 10% FBS, 0.25 U/mL of human insulin (Sigma-Aldrich Corp., St. Louis, MO, USA), 1× MEM (1 ml amino acids/100 mL of media) of non-essential amino acids (HyClone), 1 μg/ml puromycin and grown at 37°C in humidified 5% CO_2_. Cell lines were authenticated by STR profiling (IDEXX BioResearch, Columbia, MO, USA). All cell lines test negative for mycoplasma.

### Construction of stable cell lines

OVCAR3 and KURAMOCHI cell lines endogenously produce high levels of SUSD2. *SUSD2* shRNA-expressing lentiviral particles (pLKO.1 vector, MISSION^™^ shRNAs, Sigma-Aldrich Corp.) were used to generate stable SUSD2 knock-down (KD) cell lines. A non-targeting (NT) shRNA sequence was used as a control. (Sequences: SUSD2 sh1, CCGGGACGATCATTTCTGCAACTTTCTCGAGAA AGTTGCAGAAATGATCGTCTTTTTTG; SUSD2 sh2, CCGGCATCTACTTCCACTGTGACAACTCGAGTT GTCACAGTGGAAGTAGATGTTTTTTG; SUSD2 sh4, CCGGCCAAATACTCACGGCTCTAATCTCGAGATTA GAGCCGTGAGTATTTGGTTTTTTG; and NT control, CCGGGACGATCATTTCTGCAACTTTCTCGAGAA AGTTGCAGAAATGATCGTCTTTTTTG). Cells were infected according to manufacturer’s instructions and selected with 1 μg/ml puromycin. Stable clones were selected for further study based on the extent of SUSD2 knock-down determined by flow cytometry. To further enrich the OVCAR3 cell population for low SUSD2 levels, a FACSJazz fluorescence-activated cell sorter was utilized.

### Flow cytometry

All HGSOC cells lines were assessed for cell-surface levels of SUSD2 as previously described [6]. For experiments characterizing the expression of *SUSD2* in stable cell lines, HGSOC cells were stained with fluorescently conjugated primary antibodies. Three biological replicates were performed for all flow cytometry experiments using stable cell lines. The mouse monoclonal anti-SUSD2 antibody was purchased from BioLegends (San Diego, CA, USA), Cat # 327408.

### Generation of HGSOC OVCAR3 mouse model

All animal experiments were approved by the IACUC at Sanford Research (Sioux Falls, SD, USA). Sanford Research has an Animal Welfare Assurance on file with the Office of Laboratory Animal Welfare (A-4568-01) and is a licensed research facility under the authority of the United States Department of Agriculture (46-R-0011). Female athymic nu/nu mice (Charles River Laboratories, Wilmington, MA, USA) ages 6–8 weeks were housed with free access to food and water.

A total of 36 female mice received intra-peritoneal (i.p.) injections of 8 × 10^6^ OVCAR3 cells (OVCAR3-NT cells, *n* = 16; OVCAR3 sh2 cells, *n* = 16) resuspended in 500 μl of serum-free DMEM (Atlanta Biologicals, Flowery Branch, GA, USA), or serum free DMEM without OVCAR3 cells (control group; *n* = 4). Mice were separated into two separate experimental arms: *Experimental Arm 1* and *Experimental Arm 2* (Figure 2). Both experimental arms contained 3 groups of mice: OVCAR3-NT mice (*n* = 8), OVCAR3 sh2 mice (*n* = 8), and control mice (*n* = 2). HGSOC disease progression was monitored in all mice based on bi-weekly measurements of body weight, abdominal girth, and weekly analysis of tumor burden via ultrasound biomicroscopy (UBM; Vevo2100; Visual Sonics).

Mice in *Experimental Arm 1* were sacrificed at 14 weeks post-inoculation of OVCAR3 cells/serum free DMEM and analyzed for metastasis. Mouse tumor, ovaries, spleen, liver, omentum, and pancreas were harvested, fixed in 10% formalin and paraffin-embedded for IHC analysis. For OVCAR3-NT/sh2 mice in *Experimental Arm 2*, survival time reflects the time required for mice to reach endpoint criteria, defined by several variables including tumor ulceration, weight loss/gain exceeding 25% of control mice, weight exceeding 35 g, anorexia, and abdominal girth exceeding 85 mm in circumference. Mouse tumor and organs were harvested and prepared for IHC as described for mice in *Experimental Arm 1*.

### Ultrasound imaging

Mouse ascites fluid formation and tumor growth was monitored using UBM (Vevo2100; Visual Sonics). Prior to imaging, mice were anesthetized with 1.5% isoflurane in oxygen and restrained on a heated stage (37°C) using surgical tape. Anesthesia was maintained during imaging using 1–2% isoflurane in oxygen administered via a nose cone. Warmed ultrasound gel (Parker Laboratories) was applied to the abdomen of mice. An ultrasound transducer was used to scan 4 regions of each mouse abdomen, capturing the entire peritoneal cavity of all mice (Figure 4D). Images and video recordings were obtained for later data analysis.

### Immunohistochemical staining and scoring of samples

IHC analysis of OVCAR3 spheroids, as well as the mouse tissues were analyzed by IHC as described previously [6]. The BenchMark^®^ XT automated slide staining system (Ventana Medical Systems, Inc., Tucson, AZ, USA) was used for the optimization and staining of all antibodies. The Ventana iView DAB detection kit was used as the chromogen, and the slides were counterstained with hematoxylin. Omission of the primary antibody served as the negative control (not shown). The polyclonal anti-SUSD2 antibody was purchased from Prestige Antibodies Sigma-Aldrich Corp., Cat #HPA004117. The secondary antibody was purchased from Jackson ImmunoResearch Labs, Cat #111065144.

IHC staining of the mouse tissues was reviewed and scored based on the percentage of epithelial cancer cells stained for SUSD2, on a scale of 0 to 9, defining the *extensiveness* of positive staining in the specific tissue analyzed, and on a scale from 1 to 4, defining the *area* of the tissue occupied in the field of view. The scoring distribution for *extensiveness* was defined as such: a score of 0: no SUSD2 staining; 1: 1–10% positive SUSD2 staining; 2: 11–20% positive SUSD2 staining; 3: 21–30% positive SUSD2 staining; 4: 31–40% positive SUSD2 staining; 5: 41–50% positive SUSD2 staining; 6: 51–60% positive SUSD2 staining; 7: 61–70% positive SUSD2 staining; 8: 71–80% positive SUSD2 staining; 9: 81–100% positive SUSD2 staining. The scoring distribution for the area of the tissue occupied in the field of view was defined as such: 1: 1–25% of the tissue was visible in the entire field of view; 2: 26–50% of the tissue was visible in the entire field of view; 3: 51–75% of the tissue was visible in the entire field of view; and 4: 76–100% of the tissue was visible in the entire field of view. Each tissue section (pancreas and omentum) from each mouse was scanned via bright field microscopy in its entirety, in which every field was given a *total score* by multiplying the *extensiveness* score by the *area* score. *Total scores* were then added up and divided by the total number of fields needed to scan the entire tissue, giving an average total score/mouse/tissue. These scores were then averaged across groups of mice (OVCAR3-NT mice, OVCAR3 sh2 mice, and control mice).

### Western immunoblot analysis

Western immunoblot analysis was performed using whole cell lysates derived from HGSOC cell lines as previously described with minor alterations [6]. Forty μg of protein lysate/cell line was analyzed. Equal loading was verified by incubating the membranes with anti-GAPDH antibody. Primary antibodies used include monoclonal mouse anti-GAPDH (GeneTex, Irvine, CA, USA), Cat #GTX627408, polyclonal rabbit anti-SUSD2 (C-term. fragment; Prestige Antibodies Sigma-Aldrich Corp.), monoclonal mouse anti-SUSD2 (C-term. fragment; Bio-Techne Corp.) Cat MAB9056, polyclonal rabbit anti-SUSD2 (N-term. fragment; AbCam technologies, San Francisco, CA, USA) Cat ab182147.

For immunoblotting performed in the characterization of *SUSD2* expression in stable HGSOC cells, detection was achieved with IRDye-conjugated secondary antibodies (LI-COR Biosciences) and imaged using the infrared Odyssey system; 800CW donkey anti-mouse antibody, and a 680RD donkey anti-rabbit antibody. Experiments were performed using three biological replicates for each cell line.

### Statistics

Data are expressed as mean ± S. D. Where indicated, Student’s *t*-test (2-tailed) was used to compare two groups. A *P* value 0.05 or less is considered statistically significant. Kaplan-Meier analysis was used to assess survival and the Log-rank test was used to compare the survival distributions to determine statistical significance. Center values shown represent median values. Data sets subjected to pairwise comparisons performed utilizing Student’s *t-test* were checked for normality and variance among groups via calculating Pearson’s Coefficient of Skewness (skewness coefficients fell within a range of ± 0.5) as well as equality of variance analysis (*p*-values > 0.5).

## SUPPLEMENTARY MATERIALS


