# Supplementary material for: SUSD2 expression correlates with decreased metastasis and increased survival in a high-grade serous ovarian cancer xenograft murine model
Source: Oncotarget. 2020 Jun 16;11(24):2290–301. doi: 10.18632/oncotarget.27626 (PMC7299533; doi:10.18632/oncotarget.27626)
Supplement: Supplementary file 1 [file oncotarget-11-2290-s001.pdf]

SUSD2 expression correlates with decreased metastasis and increased survival in a high-grade serous ovarian cancer xenograft murine model

SUPPLEMENTARY MATERIALS

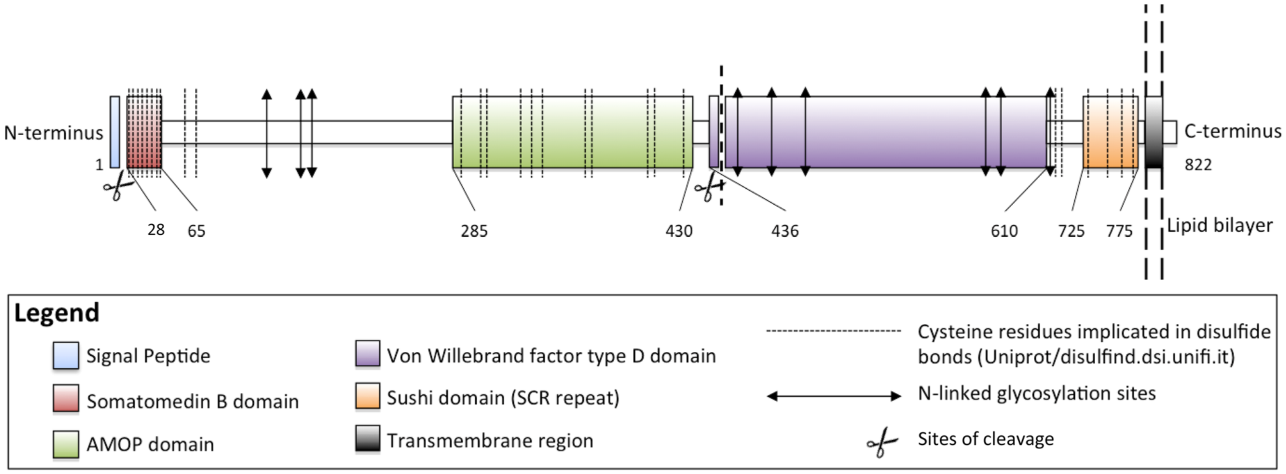

Supplementary Figure 1: Protein structure of *SUSD2*. *SUSD2* is located on Chromosome 22 and encodes a type I transmembrane protein. SUSD2 is composed of 822 amino acids and contains 4 functional domains, many of which are implicated in cell-cell adhesion. The protein has nine predicted glycosylation sites (black lines with arrows) and is cleaved at the GDPH sequence within the von Willebrand factor type D domain, yielding two fragments of similar sizes. SUSD2 contains several cysteine residues, many of which are implicated in potential disulfide bond formation (dashed black lines).

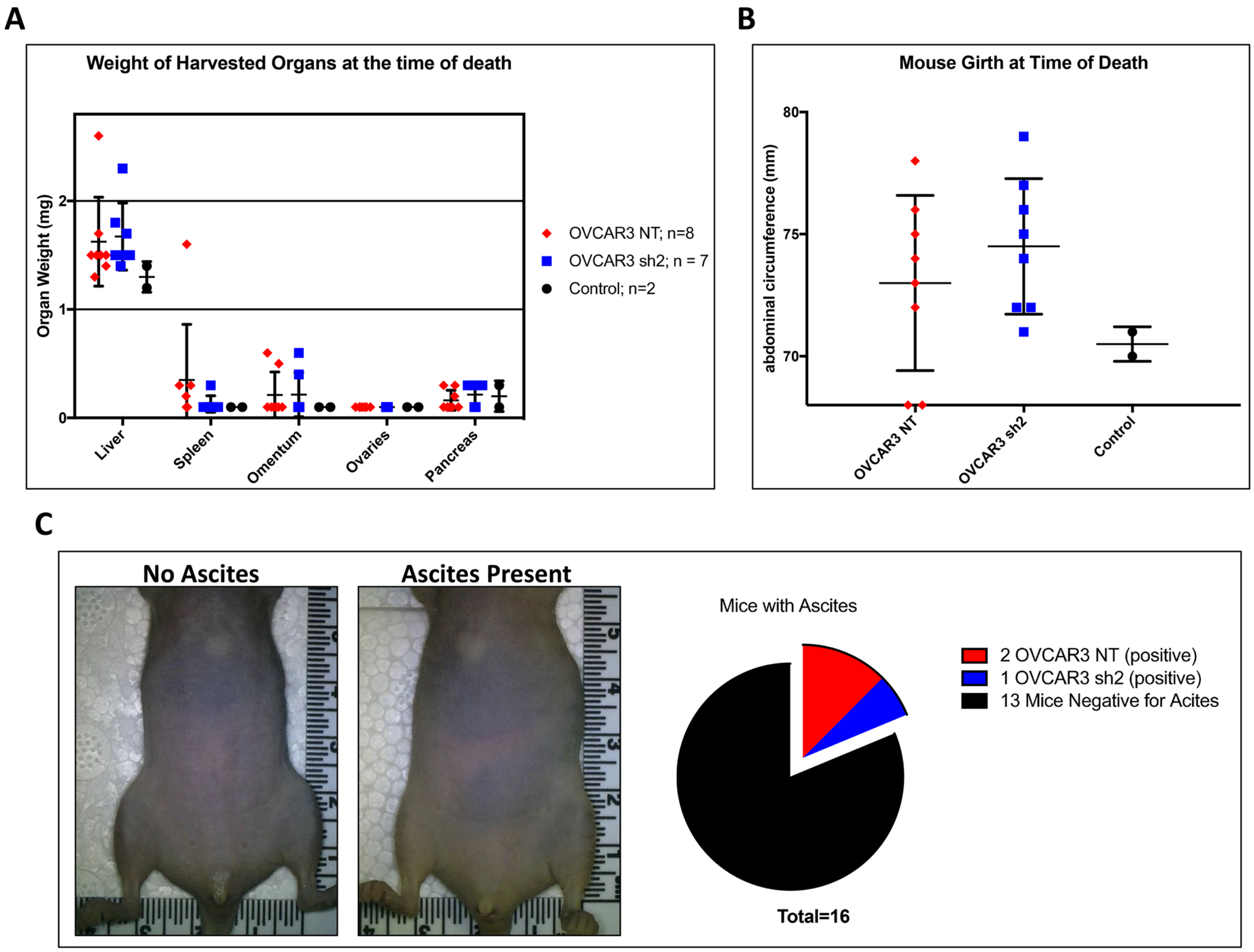

Supplementary Figure 2: Additional measurements of HGSOC progression in mouse model: organ weight, mouse girth, presence/absence of ascites. (**A**) Murine organ weight at initial time of sacrifice. (**B**) Abdominal girth at initial time of sacrifice. (**C**) Status of ascites fluid in the peritoneal cavity of mice at initial time of sacrifice. Mice imaged as seen to the left indicate a healthy abdomen (left picture) in contrast to one with ascites fluid trapped within the peritoneal cavity (right picture). The pie chart illustrates the number of mice from each experimental group (OVCAR3-NT/sh2 mice, and control mice) that were positive for ascites fluid at the initial time of sacrifice.

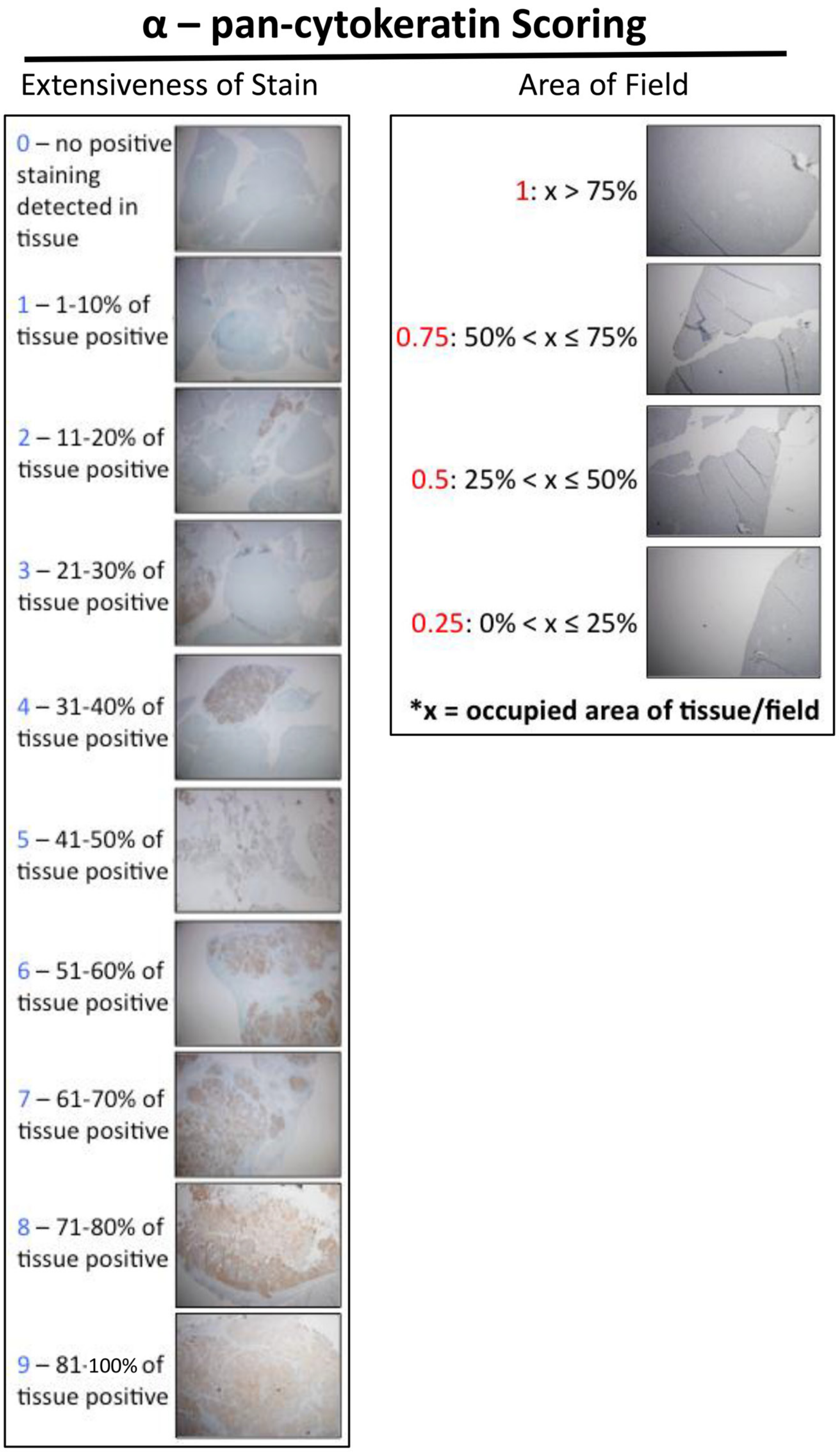

Supplementary Figure 3: Immunohistochemical scoring of pancreatic metastases. Anti-pan cytokeratin antibody was utilized to stain tumor cells within the tissue. The panel of pictures on the left (labeled "Extensiveness of Stain") shows representative images of a typical field of view under bright field microscopy post IHC staining. Extensiveness of positive staining within the tissues is indicated at the bottom of each image. The panel of pictures on the right (labeled "Area of Field") shows representative images of area occupied by pancreatic tissue. Each tissue section of pancreas harvested from mice was scored by both extensiveness (blue numbers in left column) and area of coverage (red numbers in right column) to quantitate the total amount of cancer cell infiltrate for each mouse studied.

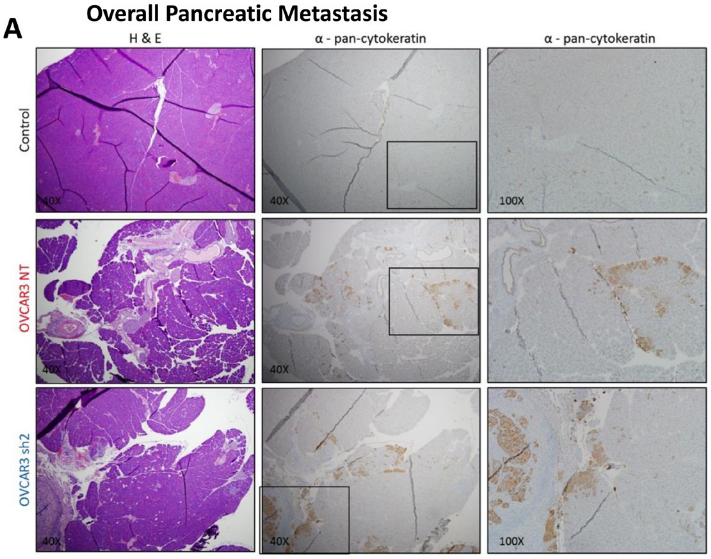

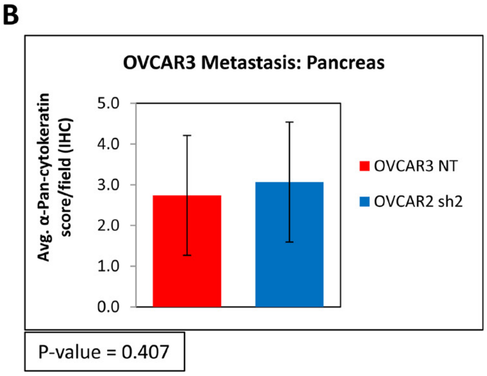

C

	OVCAR3 NT	OVCAR3 sh2
Total Number of Nodules	10	19

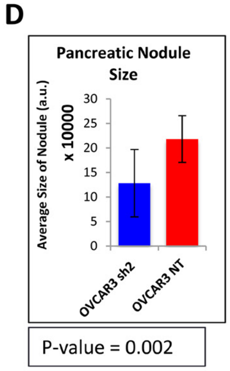

Supplementary Figure 4: Analysis of pancreatic metastasis in mouse model at time of death. (**A**) Representative images of H&E staining and pan-cytokeratin staining from each group of mice are shown at varying magnifications. Black boxes outline areas of an image that were viewed under higher magnification and reimaged (right column). (**B**) Quantification of pancreatic cancer cell infiltrate. Scoring of each mouse from each cohort (OVCAR3-NT mice are shown in red and OVCAR3 sh2 mice are shown in blue; *n* = 8) is depicted in the graph as a function of percent of positive staining/field of view. (**C**) Total number of gross tumors isolated from the pancreas. The table shows the total number of gross tumors (visible to the naked eye) harvested from the pancreas of each cohort of mice (*n* = 8). (**D**) Average size of pancreatic tumors.

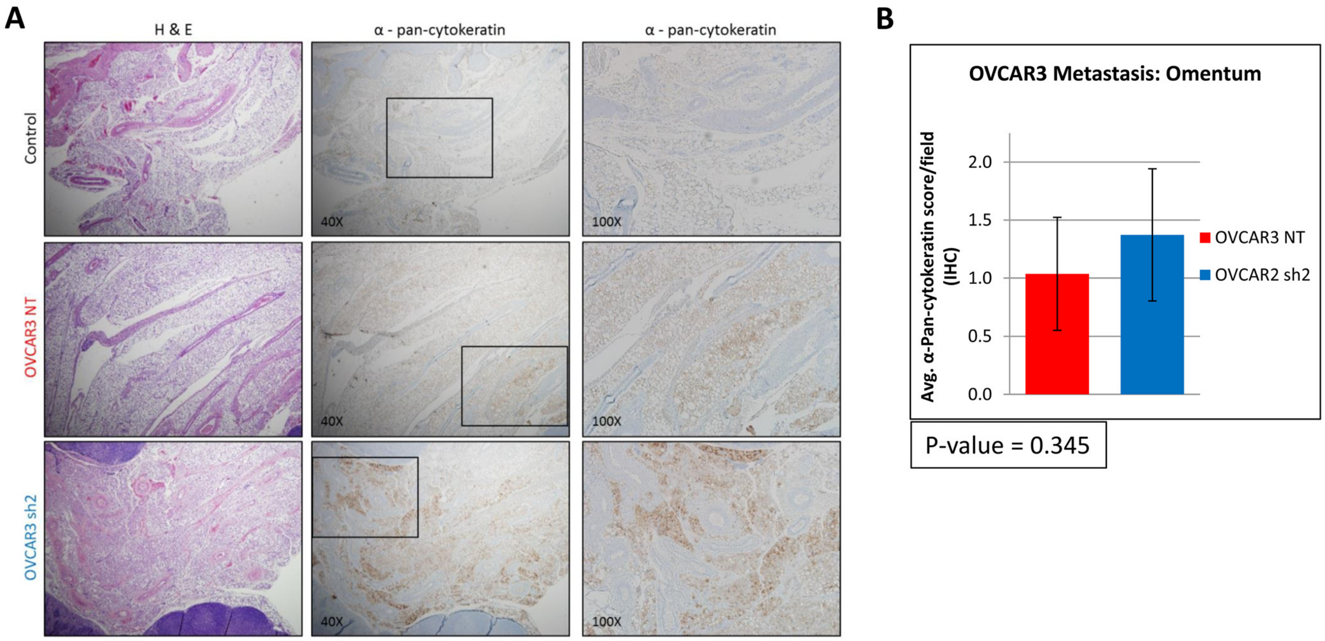

Supplementary Figure 5: Analysis of omentum metastases in mouse model at time of death. (**A**) Representative images of H&E staining and pan-cytokeratin staining from each group of mice are shown at varying magnifications. Black boxes outline areas of an image that were viewed under higher magnification and reimaged (right column). Black numbers displayed over positive pan-cytokeratin staining indicate a nodule within the omentum. (**B**) Quantification of nodules in the omentum. The total number of nodules from each mouse (*n* = 8) is depicted in the graph.
